# How genome editing changed the world of large animal research

**DOI:** 10.3389/fgeed.2023.1272687

**Published:** 2023-10-11

**Authors:** Konrad Fischer, Angelika Schnieke

**Affiliations:** Chair of Livestock Biotechnology, School of Life Sciences Weihenstephan, Technical University of Munich, Freising, Germany

**Keywords:** gene editing (CRISPR/Cas9), transgenic livestock, CRISPR/Cas9-mediated genome editing, Cpf1 (Cas12a), livestock, SCNT

## Abstract

The first genetically modified large animals were developed in 1985 by microinjection to increase the growth of agricultural livestock such as pigs. Since then, it has been a difficult trail due to the lack of genetic tools. Although methods and technologies were developed quickly for the main experimental mammal, the mouse, e.g., efficient pronuclear microinjection, gene targeting in embryonic stem cells, and omics data, most of it was—and in part still is—lacking when it comes to livestock. Over the next few decades, progress in genetic engineering of large animals was driven less by research for agriculture but more for biomedical applications, such as the production of pharmaceutical proteins in the milk of sheep, goats, or cows, xeno-organ transplantation, and modeling human diseases. Available technologies determined if a desired animal model could be realized, and efficiencies were generally low. Presented here is a short review of how genome editing tools, specifically CRISPR/Cas, have impacted the large animal field in recent years. Although there will be a focus on genome engineering of pigs for biomedical applications, the general principles and experimental approaches also apply to other livestock species or applications.

## Introduction

### The lack of tools for precise genome modification

Pronuclear DNA micro-injection has been possible in livestock since the 1990s (Hammer et al., 1985), but it was extremely inefficient in pigs (<1% of piglets born) due to the pigmented oocyte. Although the percentage was higher for other livestock species (>5%), it presented a major obstacle, considering the number of large animals required, the husbandry costs, the generation time, and unpredictable expression levels due to position effects. For both practical and animal welfare reasons, reducing the number of experimental animals to one or two founder animals with desired expression levels was an essential goal.

The first breakthrough came with the development of somatic cell nuclear transfer (SCNT) and the possibility to pre-select genetically modified cell clones (Kurome et al., 2015; Wilmut et al., 2015). Importantly, SCNT also enabled, for the first time, the generation of livestock species with targeted genetic modifications, including gene knockouts (Fischer et al., 2020), conditional targeting (Li et al., 2015), and gene placements (Rieblinger et al., 2018). However, it still had considerable drawbacks. In the absence of functional ES cells, gene targeting had to be carried out in somatic cells with limited homologous recombination capability. This restricted targeting experiments to genes that were expressed, enabling the use of promoter-trap vectors to significantly improve the selection of correctly targeted cell clones. If animals with multiple genetic modifications were required, this had to be achieved by breeding or serial nuclear transfer, which was time- and resource-intensive. Most recently, pluripotent stem cells have been isolated from pigs, cattle, and sheep (Kumar et al., 2021). However, so far, no efficient generation of chimeric animals capable of germline transmission has been reported. In addition, one could argue that with the development of CRISPR/Cas, it is no longer required.

### A new era for genome engineering in livestock

Previous gene-editing methods such as zinc-finger nucleases or transcription activator-like effector nucleases (TALENS) have all been used successfully for the generation of gene-edited (GE) livestock (Rémy et al., 2010; Flisikowska et al., 2011; Hauschild et al., 2011; Luo et al., 2014; Proudfoot et al., 2015; Carlson et al., 2016; Wang et al., 2016), but they were cumbersome or costly to generate. The fundamental breakthrough for livestock editing was triggered by CRISPR/Cas. Its RNA-based DNA recognition combined with Cas nucleases has achieved an unprecedented level of efficiency and enabled multiplexed mutations. This, in addition to the simplicity of design and low-cost production, made it the ‘system of choice’ for genetic engineering, especially in livestock. The following examples outline some of the difficulties encountered with large animal genetic engineering and the solutions provided by genome editing.

### Gene placement

Random gene insertion by either pro-nuclear DNA microinjection or transfection of cells has two main disadvantages: the position effect and the possibility of insertion mutagenesis. Although the latter is relatively rare, the position effect is a serious drawback when it comes to livestock. As a rule of thumb, at least five transgenic founder animals are required to obtain one that expresses the transgene at a desired level and—if required—in a tissue-specific manner. Pro-nuclear DNA microinjection yields a low number of transgenics, and the number of offspring for most large animal species is limited to one or two, with a pregnancy duration of 5 or 9 months (sheep and cow). It is evident that large numbers of animals are required at considerable costs. The first improvement came with SCNT, where if the embryo was reconstituted with a genetically modified cell, then the cloned offspring must be transgenic. This alone significantly reduced the number of experimental animals. Prior determination of transgene expression, however, is not always possible. It depends on the choice of the promoter and the somatic cell type used.

To ensure expression, a transgene can be placed at a locus known to support abundant ubiquitous expression, ideally at a ‘safe harbor’ dispensable for normal physiology and development. One such locus is porcine *ROSA26*, and the insertion of xeno-relevant transgenes at the locus resulted in cloned piglets with high expression levels (Rieblinger et al., 2018). To achieve this, a promoter-trap vector with large areas of homology had to be constructed. Now, the same task is achieved without the need for any regions of homology by employing CRISPR/Cas-based methods and two guide RNAs, one of which is specific for the target locus and the other excises the transgene from the plasmid backbone (homology-independent integration) (Suzuki and Izpisua Belmonte, 2018). Importantly, co-placement of a selectable marker gene is no longer required. The absence of an antibiotic resistance gene might be preferable for some applications, for example, xenotransplantation or GE livestock for food production. Assessing this approach for the porcine *ROSA26* locus, we obtained placement efficiencies of nearly 40%. Next, we tested if this method also enabled gene insertions at loci not expressed in the cells used for SCNT and where all traditional gene targeting attempts had previously failed, i.e., placement of Cre-recombinase under the control of an endogenous promoter (*PTF1*) for tissue-specific pancreatic expression (Kalla et al., 2021). It also allowed the simultaneous addition of (multiple) xeno-relevant transgenes into the intergenic region of a pre-existing transgene locus (Fischer et al., 2016). Similar approaches have also been carried out for agricultural applications, for example, for the reconstitution of uncoupling protein 1 (UCP1) in pigs, which was lost during evolution and is responsible for brown adipose tissue-mediated thermogenesis (Zheng et al., 2017). Without genome editing, none of this would have been possible.

### Gene inactivation and precision excision

**FIGURE 1:**
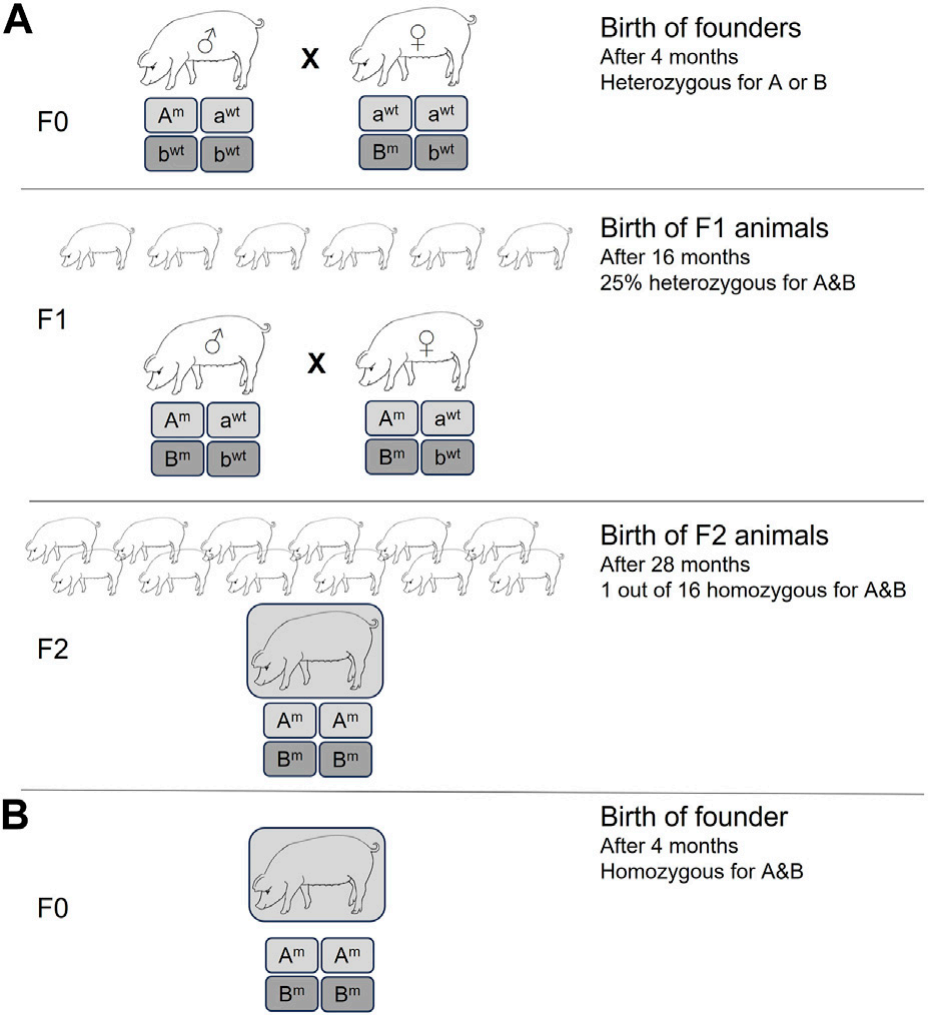
Generation of homozygous double knockout pigs by **(A)** traditional methods, resulting in heterozygous knockout founder pigs. Homozygous double knockout pigs had to be generated by subsequent breeding for at least two generations, resulting in a large number of animals with a non-desired genotype. Minimum time required is 28 months. **(B)** Homozygous double knockout pigs generated by CRISPR/Cas methods. Reduction in time (2 years) and number of experimental animals.

The CRISPR/Cas9 system has achieved unprecedented levels of efficiency for gene inactivation for nearly all species, including livestock (Ni et al., 2014; Niu et al., 2017; Fischer et al., 2020). In most cases, unless a donor DNA template is used, the DNA double-strand breaks will be repaired by non-homologous end-joining (NHEJ), causing insertions and deletions (INDELs) at the cut site that can disrupt the open reading frame, resulting in gene inactivation. Importantly, it allows for simultaneous inactivation of multiple genes by simply using more than one target-specific guide RNA. Previously, this had to be performed either by extensive breeding strategies or by serial nuclear transfer, e.g., inactivation of a single allele of gene A, followed by SCNT, re-isolation of somatic cells, targeting the second allele of gene A, and SCNT and isolation of cells to target gene B (Kuroiwa et al., 2004). Using CRISPR/Cas9, pigs with homozygous knockouts of two (Fischer et al., 2016), three (Estrada et al., 2015), or even four (Fischer et al., 2020) different endogenous genes have been generated in a single experiment. If the genome-edited pig is generated by SCNT, then simple selection of the edited cells via magnetic beads can result in efficiencies of >90% for multi-gene inactivation (Fischer et al., 2020). The pig now holds the record for the highest number of simultaneously inactivated gene copies: 25 copies of porcine endogenous retroviruses (Niu et al., 2017). The time and manpower savings, and the benefits to animal welfare (3R) cannot be underestimated. This is demonstrated in Figure 1 for the generation of pigs homozygous for just two gene knockouts. It is easy to envisage the timeline needed for the homozygous knockout of four genes, or if such an experiment would be attempted not in pigs, which have large litters and a generation time of about 1 year, but in cows with single offspring and generation time closer to 3 years.

Complex genome editing approaches, where a pre-selection of the correct targeting event is advisable, are generally carried out in somatic cells, followed by nuclear transfer. Simple knockouts or excision of DNA sequences can efficiently be achieved *in vivo* in the fertilized embryo (Hai et al., 2014; Winogrodzki et al., 2023) without any adverse effect on the porcine blastocyst development or sex ratio (Whitworth et al., 2017). For example, to generate a porcine model for inflammatory bowel disease (IBD), two guide RNAs were designed to delete the adenosine–uracil-rich element (ARE) within the 3’ UTR region of the porcine *TNF* gene. Genome editing was carried out in *in vitro-*produced embryos, and of the resulting 10 piglets, seven had a precise excision of the ARE element either on one or both alleles (Hai et al., 2014; Winogrodzki et al., 2023). One drawback of editing the early embryo is the risk of a mosaic genotype, which requires additional breeding.

Although the aforementioned examples are for biomedical models, the same applies to agricultural applications, for example, to improve animal health by inactivating viral receptors (Yang et al., 2018) or animal welfare, e.g., hornless cattle (Carlson et al., 2016). Livestock traits are rarely monogenic, and methods to modify multiple loci simultaneously would be advantageous and could increase genetic gains compared to ‘conventional’ genomic selection (Hickey et al., 2016).

### *In vivo* editing

In addition, in *in vivo* germline editing, as mentioned previously, there is considerable interest in editing specific organs or tissues in adult animals, e.g., as a model for therapeutic GE approaches in patients or to circumvent the need for generating new pig models with germline mutations, e.g., cancer models. This requires simultaneous delivery of the CRISPR components, for example, by viral vectors, which is hampered by their limited cargo capacity. Equivalent to mouse models (Platt Randall et al., 2014), Cas9 transgenic livestock species have been generated, which require the delivery of guide RNAs only, and functionality has been exemplified for cardiac- and cancer-relevant genes (Wang et al., 2017; Rieblinger et al., 2021).

In addition to its large size, Cas9 has another disadvantage as its guide RNAs are ubiquitously expressed from polymerase III promoters, while guide RNA expression for Cas12a can be driven by tissue-specific polymerase II promoters. Cas12a has a further advantage, which is the efficient processing of concatenated trans-activating (cr) RNA arrays (Jiang et al., 2019). However, the pigs we generated with the Cas12a gene placed at the *ROSA26* locus showed a very low editing efficiency compared to the equivalent Cas9 pigs. This is in contrast to a recently published mouse model (Dong et al., 2023), and the use of Cas12a variants with enhanced activity (Huang et al., 2022) might be required for the pigs. However, in parallel to the advancement of genome editing tools, the delivery methods are also continuously improving, e.g., nanoblades (Mangeot et al., 2021) or molecular syringes (Kreitz et al., 2023; Ledford, 2023), so that delivery of Cas proteins might not be an obstacle in the future.

However, there might be alternative uses for Cas transgenic pigs. They could improve the generation of edited pig lines, as shown in a recent publication for mice. Maternally expressed Cas9 enabled the production of GE mice with a higher efficiency, lower mosaicism, and multiplexing capability (Sakurai et al., 2020).

### The expanding toolbox

Few areas of research have seen such rapid development as genome editing. Improvements in Cas9, such as xCas9 or saCas9, offer smaller Cas9 molecules with a broader range of PAM sequences and higher DNA sensitivity (Hu et al., 2018). Alternatives to Cas9, such as Cas12a, can target T-rich motifs without the need for tracrRNA, thereby improving genome editing applications for the detection of transcriptional variations and base editing (Zetsche et al., 2015). Both cytosine base editors (CBEs) and adenine base editors (ABEs) have been developed. The newer systems are based on Cas-nickase combined with a base-modifying enzyme (Porto and Komor, 2023) to efficiently generate a single-base polymorphism, introducing or correcting a point mutation. It can also be multiplexed (Yuan and Gao, 2022), possibly enabling a polygenic alteration in livestock in the near future. The Cas14 protein is reported to have advanced genome editing efficiencies, capable of targeting ssDNA without the need for a PAM motive to perform transcriptional regression and base editing (Harrington et al., 2018; Hillary and Ceasar, 2023). Alternative to editing the genome, the Cas13 nuclease offers the possibility to alter the transcriptome and, thus, to treat genetic diseases without intervening permanently with the genome. The Cas13 protein is also used for diverse applications such as imaging, base editing, and detection of transcriptional variations (Cox et al., 2017; Xu et al., 2021).

### Outcomes to be considered

CRISPR/Cas9 might be the only genetic engineering technology where efficiency can be a drawback, i.e., if only one allele of a gene should be inactivated and most samples analyzed have a mutation on both alleles. Similarly, when editing is carried out in the early embryo, mosaicism can occur. When working with mice, this is quickly bred out, but for livestock, it could mean a 1- to 3-year delay, depending on the species. Using homology-independent integration, to place transgenes or insert DNA fragments into the genome, can result in the integration of concatemers. This may be advantageous if additional copies improve transgene expression. If this is not the goal, then selection for the correct single copy insertion is required.

A major concern is possible off-target effects. These might be restricted to small INDELs in non-target regions of the genome, but multiplex editing can lead to larger chromosomal rearrangements. Methods to assess off-target events and prediction software are continuously improving. The latter may even include deep learning approaches to consider chromatin organization states and epigenetic modifications (Chuai et al., 2018; Listgarten et al., 2018). Proofreading optimizations of Cas9 itself, e.g., enhanced Cas9, high-fidelity Cas9, or hypaCas9, show increased fidelity and significantly reduced off-target activities (Chen et al., 2017). In addition, methods such as GUIDE-tag enable the incorporation of short biotinylated DNA double-strand oligonucleotides into Cas9-mediated DNA double-strand breaks to detect non-predicted off-target sites *in vivo* (Liang et al., 2022). Avoiding off-targets is especially important if GE livestock is intended to enter the food chain or for xeno-organ transplantation. At the same time, one has to keep in mind that, on average, any animal born will carry about 70 novel mutations.

## Conclusion

Genome editing has a larger effect on modifying the genome of livestock than for any other experimental animal such as the mouse. In the years before GE, the generation of a single gene-targeted large animal was published per year. This has increased more than twenty-fold, covering both biomedical and agricultural applications, and includes ever more complex genome modifications. CRISPR provides a clear advantage for animal welfare as far fewer animals are required to generate a desired genotype. However, more modified animals are being created, including species that were so far not amenable to genetic modifications. It has been a pivotal technology in bringing xeno-heart transplantation into the clinic (Griffith et al., 2022) and advance medical procedures (Uddin et al., 2020; Liu et al., 2021), and it can improve resilience, fertility, growth, and welfare of agricultural animals. The first approvals for marketing genome-edited animal products have been granted, e.g., Japan approved the sale of two types of edited fish (Author anonymous, 2022), and the FDA issued several “low-risk determinations” for the marketing of products from two genome-edited beef cattle, goat, chicken, salmon, and pigs (FDA), and others are to follow. There are still ethical and legal uncertainties, but genome editing is here to stay and will have a major impact on genetic engineering in all livestock species.
